# Effect of *Auricularia auricula* aqueous extract on survival of *Lactobacillus acidophilus La‐5* and *Bifidobacterium bifidum Bb‐12* and on sensorial and functional properties of synbiotic yogurt

**DOI:** 10.1002/fsn3.1414

**Published:** 2020-01-20

**Authors:** Azita Faraki, Negin Noori, Hassan Gandomi, Sayed Attaul Haq Banuree, Fatemeh Rahmani

**Affiliations:** ^1^ Department of Food Hygiene Faculty of Veterinary Medicine University of Tehran Tehran Iran; ^2^ Department of Pre‐clinic Veterinary Science Faculty Nangarhar University Nangarhar Afghanistan

**Keywords:** antioxidant activity, *Auricularia auricula*, probiotic, total phenol, yogurt

## Abstract

The effect of *Auricularia auricula* aqueous extract (AAE) on the survival of *Lactobacillus acidophilus La‐5* and *Bifidobacterium bifidum Bb‐12,* and on chemical and sensory properties of yogurt was investigated during 28 days of storage at 4°C. The use of 0.05% of AAE improved the survival of *L. acidophilus La‐5* and *B. bifidum Bb‐12* about 0.35 and 0.58 log CFU/g, respectively. However, AAE in 0.1% concentration enhanced the survival of *L. acidophilus La‐5* and *B. bifidum Bb‐12* about 0.43 and 0.51 log CFU/g, respectively. Moreover, 0.1% concentration of AAE drastically increased antioxidant activity and total phenolic content to 115.30 mg BHT eq./kg and 1,057.6 mg Gallic acid/kg after 28 days, respectively. Addition of AAE to the yogurt significantly decreased sensorial acceptance while increased syneresis compared to the control group (*p* < .05). In conclusion, the results of this study showed that addition of AAE improved probiotic protection and functional properties of the yogurt recommending its application in symbiotic yogurt.

## Graphical abstract

The use of *Auricularia auricula* extract in probiotic yogurt improved the viability of probiotics and increased the antioxidant activity and total phenolic content.

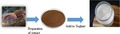

## 1 INTRODUCTION

According to the definition of FAO/WHO, probiotics can be defined as: “live microorganisms that, when administered in adequate amounts, confer a health benefit on the host” (Hill et al., 2014). In dairy fermentation, probiotics play important roles such as production of antimicrobial compounds and other metabolites, controlling gastrointestinal infections, improvement in lactose metabolism, anticarcinogenic and antimutagenic properties, cholesterol reduction, immune system stimulation, and improvement in inflammatory bowel disease (Ott, Hugi, Baumgartner, & Chaintreau, 2000; Pinto, Clemente, & De Abreu, 2009). Most probiotics are classified in the category of lactic acid‐producing bacteria and are normally consumed in the form of yogurt, fermented milk products, or other fermented foods. *Lactobacillus* and *Bifidobacterium* genera are most widely used probiotics, and various species of them are formulated in most probiotic products (Chen et al., 2017). The viability and stability of probiotics are of utmost importance during product shelf life to ensure the minimum satisfactory level at the time of consumption so as to achieve the claimed health benefits (Mattila‐Sandholm et al., 2002). Among several strategies, the use of prebiotics is well established for the improvement of probiotics viability. The definition of a prebiotic can be described as: “a substrate that is selectively utilized by host microorganisms conferring a health benefit” (Gibson et al., 2017). Inulin, fructooligosaccharide, soybean oligosaccharides, and galactooligosaccharide are the main prebiotics used in the food industry (Schrezenmeir & De Vrese, 2001). The Functional Food Center (FFC) defines functional food as: “natural or processed foods that contains known or unknown biologically‐active compounds; the foods, in defined, effective, and non‐toxic amounts, provide a clinically proven and documented health benefit for the prevention, management, or treatment of chronic disease” (Martirosyan & Singh, 2015). Dairy and probiotic products can be a category in functional foods because they provide health benefits beyond the traditional nutrition function (Granato, Branco, Cruz, Faria, & Shah, 2010; Lin, 2003). Also, dairy products are highly accepted by consumers and play an important role as carriers of probiotics and yogurt is the most common dairy product consumed around the world, and its sensory attributes have a large effect on consumer acceptability (Allgeyer, Miller, & Lee, 2010; Tuomola, Crittenden, Playne, Isolauri, & Salminen, 2001). The term synbiotic is used when a product contains both probiotics and prebiotics (Lourens‐Hattingh & Viljoen, 2001). There are a lot of mushrooms on Earth that is estimated at 140,000 species. However, only 10% of them are recognized as food and therapeutic source of which *Auricularia auricula* is the most popular mushroom in traditional medicine. Its body shape is similar to human ear and otherwise called Jew's ear, jelly ear and by a number of other common names, as like in Japan “tree jellyfish,” in China “wood ear,” and in Russia it is called “black fungus.” The brown color is well defined and the body size is between 3 and 12 cm. *Auricularia auricula* typically grows on the trunk of trees, especially on elder ones. Main components of this cultivated mushroom are ash (3.6%), protein (12.5%), fat (1.7%), total carbohydrate (66.1%), and another components like water‐soluble polysaccharide, cellulose, chitin, pectin, amino acid, and mineral element contents (Kadnikova, Costa, Kalenik, Guruleva, & Yanguo, 2015). It has been shown that the mushroom has antitumor activity (Ma, Wang, Zhang, Zhang, & Ding, 2010) and regulates blood pressure (Acharya, Samui, Rai, Dutta, & Acharya, 2004), hypocholesterolemic activity, hypolipidemia activity, enhancing immunity, lowering blood glucose, anti‐aging (Zhang, Wang, Zhang, & Wang, 2011), antiviral activity (Nguyen et al., 2012), anticoagulant activity (Yoon et al., 2003), anti‐inflammatory activity (Damte, Reza, Lee, Jo, & Park, 2011), and antimicrobial activity (Yu & Oh, 2016). As far, we know there is no study concerning prebiotic and antioxidant activity of this mushroom in yogurt. Hence, the present study was aimed to investigate the probiotic, antioxidant, physicochemical, and sensory properties of synbiotic yogurts with *Auricularia auricula* extract during refrigerated storage.

## 2 MATERIALS AND METHODS

### 2.1 Preparation of microorganisms

Starter culture of yogurt containing *Lactobacillus delbreukii* subsp. *bulgaricus* and *Streptococcus salivarius* subsp. *thermophilus* and lyophilized strains of *Lactobacillus acidophilus La‐5* and *Bifidobacterium bifidum Bb‐12* were purchased from Chr. Hansen Company. The probiotic bacteria were activated by inoculation in de Man–Rogasa–Sharpe (MRS) broth (Merck) and incubation at 37°C for 24 hr and second subcultures were prepared in the same way for 48 hr at 37°C. The biomass in late log phase was harvested by centrifugation (Eppendorf AG) at 10,000 *g* for 10 min at 4°C and washed twice in sterile physiological saline solution. Afterward, 2 ml sterile physiological saline solution was added to the tubes in order to produce probiotic suspension. Then, the produced suspension was vortexed to allow bacterial deposition to be uniformly distributed. Finally, by using the spectrophotometer (BECKMAN), inoculum was adjusted to the required concentration of 10^9^ CFU/ml (Noori, Hamedi, Kargozari, & Shotorbani, 2017).

### 2.2 Preparation *Auricularia auricula* aqueous extract

A total of 500 g of *Auricularia auricula* was supplied from Nur city (north of Iran) and after authentication by mycology research center, Faculty of Veterinary Medicine, University of Tehran, was placed at room temperature for drying. Then, dried mushrooms were milled using grinder (IKA M20 universal) and transferred into a Simax glass and mixed with 2 liters of water. The mixture was placed on shaker (T&N, China) for 24 hr. Hereafter, the mixture was filtered through Wathman #2 filter paper. The extract was concentrated with rotary evaporator (STRIKE 100, Steroglass) and dried in oven at 40°C. Finally, the prepared extract was stored in a dark glass container at 4°C until its intended use (González‐Palma et al., 2016).

### 2.3 Yogurt preparation

Pasteurized cow's milk containing 3.5% fat and 3.5% protein with pH 6.7 was used for yogurt production. The final milk nonfat dry matter content was standardized to 11% (w/w) with nonfat dried milk. Then, milk was heated to 90–95°C for 5 min and rapidly cooled to 42°C and 2% (w/v) starter culture and 1% of probiotic suspension was added. Four groups of yogurt samples were prepared: yogurt without AAE and probiotic as control, yogurt containing probiotic bacteria, yogurt containing probiotic bacteria and 0.05% of AAE, and yogurt containing probiotic bacteria and 0.1% of AAE. All mixtures were poured into 100 ml sterile glass containers in triplicate fashion and incubated at 42°C until pH was reached to 4.4–4.5. The produced yogurts were then stored at 4°C for 28 days (Bertrand‐Harb, Ivanova, Dalgalarrondo, & Haertllé, 2003). Independent experiment was repeated twice.

### 2.4 Enumeration of probiotic bacteria

Enumeration of probiotic bacteria was performed by the standard enumeration techniques using tenfold serial diluting prepared in 0.1% (w/v) buffered peptone water (Merck). The method used for enumeration of probiotic bacteria, along with some modifications. *Lactobacillus acidophilus La‐5* was cultured in MRS‐bile agar at aerobic condition and *B. bifidum Bb‐12* was cultured in MRS agar (Merck) containing 0.3% sodium propionate (Sigma‐Aldrich) and 0.05% L‐cysteine (Sigma‐Aldrich) at anaerobic conditions. The cultured probiotics were then incubated at 37°C for 48 hr. The number of viable cells of probiotics was reported as CFU/g (Van de Casteele et al., 2006; Vinderola & Reinheimer, 1999).

### 2.5 pH and titratable acidity

The pH of yogurts was measured using a digital pH meter (Jenway 3320). For titratable acidity determination, 10 g of yogurt was mixed with 90 ml of distilled water and titrated with 0.1 M sodium hydroxide using phenolphthalein as indicator. The titratable acidity was calculated using the following equation, and results were expressed as gr lactic acid/L of yogurt (Zainoldin & Baba, 2009).

$$\text{Titratable acidity}=\;\left(V\;\times \;0.009\;\times \;100\right)/m$$

V = volume of NaOH used to neutralize the lactic acid.m = Sample weight (g).

### 2.6 Antioxidant activity

The method used for this spectrophotometric assay was done using the stable radical DPPH as a reagent and along with some modifications. A total of 100 μL of the sample was mixed with freshly prepared DPPH solution (0.004% (w/v) in methanol solution) and allowed to react for 30 min at room temperature. DPPH solution without bacteria was used as control. DPPH scavenging activity was monitored by decrease in absorbance at 517 nm, which was calculated using the following formula:

$$I\%\;=\left(A_{\text{blank}}-\;A_{\text{sample}}/\;A_{\text{blank}}\right)\;\times \;100$$

where *A*_blank_ is the absorbance of control (containing all reagents except sample), and *A*_sample_ is the absorbance of the sample (Burits & Bucar, 2000).

### 2.7 Total phenolic content

Five grams of each sample was mixed with distilled water and centrifuged for 15 min at 10,000 *g*. A total of 0.1 ml of supernatant was mixed with Folin–Ciocalteu reagent (1:2 deionized water) and sodium carbonate (2%). Subsequently, the samples were incubated in a dark place at 25°C for 2 hr and absorbance was measured at 750 nm using a spectrophotometer (BECKMAN). Results were expressed as mg Gallic acid equivalent/g of kg yogurt (Agbor, Vinson, & Donnelly, 2014; Vasco, Ruales, & Kamal‐Eldin, 2008).

### 2.8 Syneresis

The method used for syneresis test, along with some modifications. Twenty grams of each yogurt sample was centrifuged at 10,000 *g* for 20 min at 10°C. After centrifugation, the supernatant was removed and the pellet was collected and weighed. The percent of syneresis was calculated as follows:

$$\text{Syneresis}\;=\;\left(W_{t}/\;W_{i}\right)\;\times \;100$$

where* W_t_* is weight (g) of the pellet and *W_i_* is the weight (g) of the sample (Sahan, Yasar, & Hayaloglu, 2008).

### 2.9 Sensory assessment

Seven panelists were selected from the Department of Food Hygiene and Quality Control, University of Tehran, on the basis of their experience in the sensory analysis. The sensory parameters (appearance, taste, texture, and overall acceptability) were assessed using a 5‐point hedonic scale as like 5 indicated like extremely, 4 for like moderately, 3 for neither like nor dislike, 2 for dislike moderately, and 1 indicated dislike extremely (Hamedi, Razavi‐Rohani, & Gandomi, 2014). Sensory examination was done at day 1 of storage.

### 2.10 Statistical analysis

The data collected in this study were analyzed using SPSS Version 20.0 for Windows. One‐way analysis of variance (ANOVA) was performed to investigate the significant difference among groups. Duncan's test was used as multiple comparison test. Statistical significance was set at *p* < .05. The results of analysis were reported as mean ± standard deviation (*SD*).

## 3 RESULTS

### 3.1 Viability of probiotics

**Figure 1:**
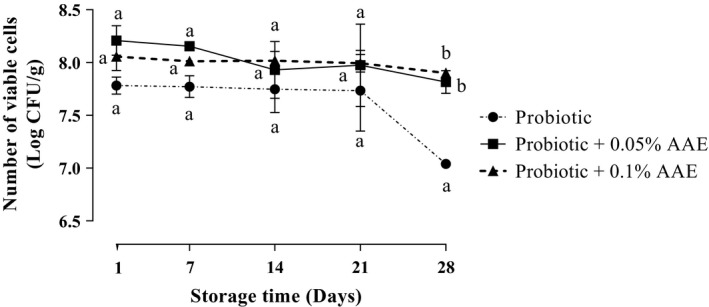
The effect of *Auricularia auricula* aqueous extract (AAE) on the viability of *Lactobacillus acidophilus* La‐5 in studied groups during 28 days of storage at 4°C. Values followed by different lower case letters at the same days are significantly different

**Figure 2:**
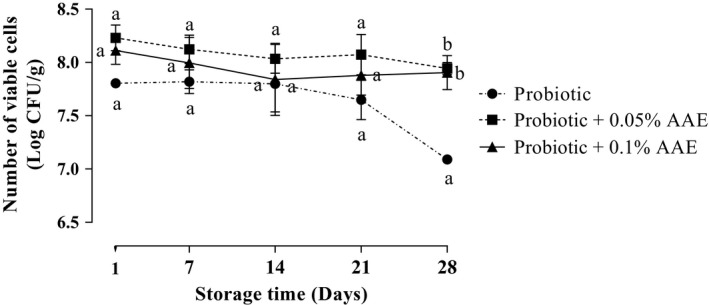
The effect of *Auricularia auricula* aqueous extract (AAE) on the viability of *Bifidobacterium bifidum* Bb‐12 in studied groups during 28 days of storage at 4°C. Values followed by different lower case letters at the same days are significantly different

The results of *L. acidophilus La‐5* and *B. bifidum Bb‐12* count in all treatments are presented in Figure 1. According to the results, there was no significant (*p* > .05) difference in *L. acidophilus La‐5* counts among studied groups during 28 days of storage at 4°C unless at day 28 which the *L. acidophilus La‐5* count of probiotic group was significantly (*p* < .05) lower than AAE containing groups. In probiotic yogurt, the log number of viable cells of *L. acidophilus La‐5* was 7.78 CFU/g in first day which was significantly (*p* < .05) decreased to 7.04 CFU/g on day 28. However, the count of *L. acidophilus La‐5* was significantly (*p* < .05) dropped from 8.21 to 7.82 in 0.05% containing yogurt after 28 days of storage. No significant difference was seen in *L. acidophilus* count between 0.05% and 0.1% AAE containing groups (*p* > .05). The results of *B. bifidum Bb‐12* count in all treatments are presented in Figure 2. In probiotic yogurt, the log number of viable cells of *B. bifidum Bb‐12* was 7.81 log CFU/g in first day which was significantly (*p* < .05) decreased to 7.09 log CFU/g on day 28. The log number of viable cells of *B. bifidum Bb‐12* was significantly (*p* < .05) dropped from 8.23 to 7.94 log CFU/g in 0.05% AAE containing yogurt during storage period. No significant difference was seen in *B. bifidum* count between 0.05% and 0.1% AAE containing groups (*p* > .05).

### 3.2 The effect of *Auricularia auricula* extract on chemical parameters in yogurt

#### 3.2.1 Antioxidant activity

**Figure 3:**
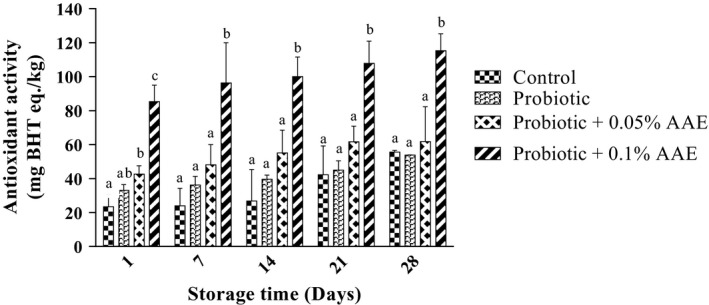
The effect of *Auricularia auricula* aqueous extract (AAE) on antioxidant activity in studied groups during 28 days of storage at 4°C. Values followed by different lower case letters at the same days are significantly different

Figure 3 depicts the results of antioxidant activity of different groups. There was a significant difference in antioxidant activity between 0.1% AAE containing group and other studied groups at all examined days (*p* > .05). The antioxidant activity was increased during the experiment since antioxidant activity of control, probiotic yogurt, 0.05%, and 0.1% AAE containing yogurts was ranged from 23.4, 33.0, 42.6, and 85.3 mg BHT eq./kg at day 1 to 55.6, 53.9, 61.7, and 115.30 mg BHT eq./kg at day 28, respectively. There was a positive and strong correlation between antioxidant activity and AAE concentration (*r* = 0.820, *p* < .05).

#### 3.2.2 Total phenolic content

**Figure 4:**
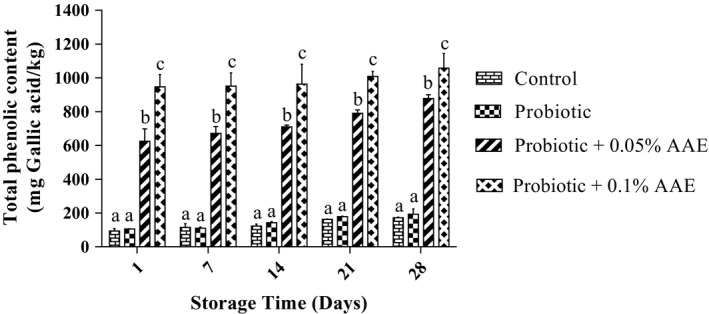
The effect of *Auricularia auricula* aqueous extract (AAE) on the total phenolic content in studied groups during 28 days of storage at 4°C. Values followed by different lower case letters at the same days are significantly different

The total phenol contents of studied groups are presented in Figure 4. No significant difference was observed between control and probiotic yogurts (*p* > .05). According to results, the total phenolic content of the studied groups was significantly increased (*p* < .05) during the storage time, since the phenolic content of control, probiotic yogurt, 0.05%, and 0.1% AAE containing yogurts varied from 94.0, 105.6, 624.4, and 946.2 mg Gallic acid/kg at day 1 to 171.5, 193.7, 877.9, and 1,057.6 mg Gallic acid/kg at day 28, respectively. Moreover, there was a positive and strong correlation between phenolic content and AAE concentration (*r* = 0.934, *p* < .05).

#### 3.2.3 pH

The results of the pH in studied groups were shown in Table 1. No significant differences (*p* > .05) were observed in pH between different groups at days 1 and 28 of study period despite of significant difference (*p* < .05) at days 7, 14, and 21. During the study period, the pH of all the studied groups was dropped significantly (*p* < .05).

**Table 1:** The effect of *Auricularia auricula* aqueous extract (AAE) on pH in studied groups during 28 days of storage at 4°C

Groups	Day				
	1	7	14	21	28
Control	4.27 ± 0.0^a^	4.08 ± 0.0^a^	4.07 ± 0.0^a^	4.07 ± 0.0^a^	3.96 ± 0.1^a^
Probiotic	4.09 ± 0.0^a^	3.87 ± 0.0^c^	3.81 ± 0.1^b^	3.84 ± 0.2^b^	3.86 ± 0.1^a^
Probiotic + 0.05% AAE	4.13 ± 0.0^a^	3.96 ± 0.0^b^	3.91 ± 0.1^ab^	3.86 ± 0.0^b^	3.92 ± 0.0^a^
Probiotic + 0.1% AAE	4.10 ± 0.0^a^	3.89 ± 0.0^a^	3.84 ± 0.0^ab^	3.79 ± 0.0^b^	3.87 ± 0.1^a^

* Values represent averages ± standard errors for duplicate experiments.

** Values followed by the same letters in each column are not significantly different at the .05 level.

#### 3.2.4 Titratable acidity

The acidity of studied groups during storage period is summarized in Table 2. There was a significant difference in acidity between groups during storage time (*p* < .05). The lowest value of acidity was related to control group which was 0.52 on day 1 and 0.79 g lactic acid/L on day 28. Although there was no significant difference between control and probiotic yogurt on first day, the acidity of probiotic yogurt was increased to 0.95 g lactic acid/L during storage period. Based on the results, the addition of AAE to yogurt resulted in increased acidity as 0.05% and 0.1% AAE enhanced the acidity from 0.76 to 1.08 and 0.81 to 1.28 g lactic acid/L, respectively.

**Table 2:** The effect of *Auricularia auricula* aqueous extract (AAE) on titratable acidity (g lactic acid/L) in studied groups during 28 days of storage at 4°C

Groups	Day				
	1	7	14	21	28
Control	0.52 ± 0.0^a^	0.54 ± 0.0^a^	0.57 ± 0.0^a^	0.65 ± 0.0^a^	0.79 ± 0.0^a^
Probiotic	0.54 ± 0.0^a^	0.68 ± 0.0^b^	0.68 ± 0.0^a^	0.70 ± 0.0^a^	0.95 ± 0.1^ab^
Probiotic + 0.05% AAE	0.76 ± 0.0^b^	0.96 ± 0.0^c^	1.02 ± 0.0^b^	1.04 ± 0.0^b^	1.08 ± 0.0^bc^
Probiotic + 0.1% AAE	0.81 ± 0.0^b^	0.99 ± 0.0^c^	1.06 ± 0.0^b^	1.14 ± 0.1^b^	1.28 ± 0.1^c^

* Values represent averages ± standard errors for duplicate experiments.

** Values followed by the same letters in each column are not significantly different at the .05 level.

#### 3.2.5 Syneresis

The results of syneresis of yogurts are presented in Table 3. There was an extensive statistically significant increase in syneresis in all treatments during storage time (*p* < .05). Generally, the syneresis of control group was lower compared to 0.1% AAE containing group. During the study period, syneresis of control, probiotic yogurt, 0.05%, and 0.1% AAE containing yogurts was increased from 39.1% to 49.5%, 40.9% to 51.3%, 43.40% to 52.9%, and 44.7% to 54.9% on day 28, respectively.

**Table 3:** The effect of *Auricularia auricula* aqueous extract (AAE) on syneresis (% w/w) in studied groups during 28 days of storage at 4°C

Groups	Day				
	1	7	14	21	28
Control	39.1 ± 0.7^a^	40.2 ± 0.7^a^	45.2 ± 0.2^a^	47.0 ± 0.8^a^	49.5 ± 0.6^a^
Probiotic	40.9 ± 0.7^ab^	41.6 ± 0.2^a^	46.8 ± 0.1^a^	47.6 ± 0.7^a^	51.3 ± 0.4^ab^
Probiotic + 0.05% AAE	43.4 ± 0.9^ab^	46.2 ± 0.8^b^	48.6 ± 0.8^a^	50.8 ± 0.9^a^	52.9 ± 0.2^ab^
Probiotic + 0.1% AAE	44.7 ± 0.6^b^	49.2 ± 0.6^b^	50.3 ± 0.6^a^	53.4 ± 0.1^a^	54.9 ± 0.9^b^

* Values represent averages ± standard errors for duplicate experiments.

** Values followed by the same letters in each column are not significantly different at the .05 level.

#### 3.2.6 Sensory assessment

**Figure 5:**
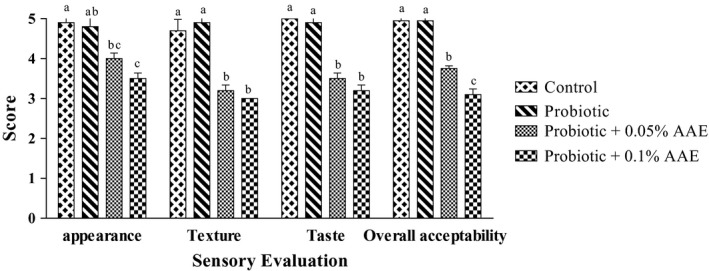
The effect of *Auricularia auricula* aqueous extract (AAE) on sensory assessment in studied groups during 28 days of storage at 4°C. Values followed by different lower case letters at the same days are significantly different

The results of sensory evaluation of different yogurts are shown in Figure 5. The control group touched the peak of sensory scores in all parameters followed by probiotic, 0.05%, and 0.1% containing yogurts. No significant difference (*p* > .05) was observed between appearance of control (4.9) and probiotic yogurts (4.8). However, addition of AAE resulted in reduction of appearance as 4.0 and 3.2 scores were recorded for 0.05% and 0.1% AEE containing yogurts, respectively. Furthermore, the sensory analysis has shown no significant difference in terms of taste and texture between control and probiotic groups while addition of AAE significantly (*p* < .05) reduced the score of aforementioned sensorial properties. The results showed that the increased concentration of AAE resulted in increased bitterness of yogurts. The overall acceptability of all produced yogurts was beyond the medium score as the lowest score was recorded for 0.1% AAE containing yogurt followed 0.05% containing AAE, probiotic, and control yogurts. The difference between control and probiotic yogurts in terms of overall acceptability was not significant (*p* > .05). However, significant difference (*p* < .05) was observed between AAE containing, and probiotic and control yogurts. Moreover, increase in the concentration of AAE caused significant difference (*p* < .05) in overall acceptability of yogurt.

## 4 DISCUSSION

### 4.1 Viability of probiotics

The prebiotic properties of *Auricularia auricula* (AA) have been studied in culture media, but few studies have been recorded in food models. Sawangwan, Wansanit, Pattani, and Noysang (2018) investigated the prebiotic properties of edible mushroom extracts in culture media and concluded that AA due to its richness in galactose and maltotriose that are indigestible carbohydrates improved the growth of *L. acidophilus* and *Lactobacillus plantarum*. Also, similar results are reported by Nowak, Nowacka‐Jechalke, Juda, and Malm (2018) as they confirmed the high polysaccharides content in various types of edible mushrooms which caused increased metabolism rate of useful bacteria such as different species of Lactobacillus. Furthermore, the results revealed that the polysaccharides of edible mushrooms significantly increased the growth of probiotic bacteria in comparison with commercial prebiotic supplementation such as inulin and fructooligosaccharides. Other studies pointed to the ability of probiotics for utilization of β‐glucan, an important polysaccharide in AA and other mushrooms (Gee, Vasanthan, & Temelli, 2007; Snart et al., 2006). Furthermore, Nasution, Rahayu, and Nasution (2018) documented the positive significant effect of different concentrations of *Auricularia polytricha* on the growth of *Lactobacillus casei*. Saman et al. (2016), found that the positive and bifidogenic effect of *Auricularia auricula* on the *Bifidobacterium animalis* as the viable cell count of probiotic at 0 hr was 4.5 log CFU/ml and at 20 hr reached to the highest level approximately 6 log CFU/ml. In another study, the log number of Lactobacillus after 0, 24, and 48 hr in the presence of polysaccharides extracted from *Auricularia auricula* was 10.99, 11, and 11.15 log CFU/ml, respectively. Likewise, the log number of Bifidobacterium during mentioned times was 10.54, 10.59, and 10.11 log CFU/ml, respectively. Additionally, the highest effect on the log number of Lactobacillus and Bifidobacterium was recorded for *Auricularia auricula* mushroom compared to commercial yeast and *Schizophylum commune* (Chaikliang, Wichienchot, Youravoug, & Graidist, 2015). According to the Vasiljevic, Kealy, and Mishra (2007) results, the addition of extracted β‐glucan from oat and barley in comparison with the control group improved the growth of *Bifidobacterium animalis* during fermentation and increased viability during storage. The addition of β‐glucan from oat to yogurt had the similar effect like inulin and maintained the probiotic cells above the lowest recommended therapeutic level (6 log CFU/ml) at the end of storage. The results also showed about 0.5 and 1 log CFU/ml decline in barley β‐glucan containing and control group yogurts after 3 weeks of storage, respectively. These results are in agreement with present study findings. According to the present study results, reduction of probiotics was about 1 log CFU/g in yogurt containing AAE after 4 weeks. Dave and Shah (1997) reported that reduction of probiotics is due to the development of harsh environmental conditions as a result of organic acid production during the storage time.

### 4.2 Antioxidant activity and total phenolic content

In the present study, the addition of 0.05% and 0.1% of AAE to yogurt increased antioxidant activity about 13.1 and 2.2 mg BHT eq./kg, respectively. Also, the addition of 0.05% and 0.1% of AAE to yogurt caused considerable increase in terms of total phenolic content about 175.9 and 33.9 mg Gallic acid/kg, respectively. This feature has been proved in various studies. There was a positive and strong correlation between phenolic content and AAE concentration (*r* = 0.807, *p* < .05) and also between antioxidant activity and AAE (*r* = 0.820, *p* < .05) as well. The antioxidant activity, phenolic, and polysaccharide content of 49 types of edible mushrooms were studied in China, and they stated that there is a positive correlation between antioxidant activity and phenolic content, which indicates phenolic compounds were main contributors of antioxidant activity of mushrooms, also reported that phenolic compounds of AA were Gallic acid, Protocatechuic acid, and p‐Hydroxybenzoic acid, and the EC_50_ values for inhibition of hydroxyl radical and inhibition of lipid peroxidation were 0.373 and 0.398 mg/ml, respectively (Guo et al., 2012). Oke and Aslim (2011) investigated the antioxidant activity and phenolic content of aqueous and methanolic extracts of AA. Their results showed high antioxidant activity for both extracts. Moreover, the results reported Gallic acid, Catechin, Hydroxybenzoic acid, and Caffeic acid as most important phenolic content. In another study, Khatua, Paul, and Acharya (2013) described mushrooms as a new potent source of natural antioxidant and the main bioactive compounds in mushroom included phenolic compounds (phenolic acids and plavonoids), tocopherols, ascorbic acid, carotenoids, and carbohydrates. Another study reported species‐dependent effect of mushroom on activity (Xu, Zhang, & Jiang, 2016). Fan, Zhang, Yu, and Ma (2007) presented that bread made from flour enriched with polysaccharides of AA had high antioxidant activity that was in turn related to β‐glucan. Another study also documented higher antioxidant activity of low molecular weight polysaccharides (2.8 × 10^4^ Da) from *Auricularia polytricha* compared to vitamin C at same concentration. Kho, Vikineswary, Abdullah, Kuppusamy, and Oh (2009) compared fresh, oven‐dried, and freeze‐dried methanolic extracts of AA in terms of antioxidant activity and total phenolic content. Based on their results, the antioxidant property and total phenolic content of freeze‐dried extract were significantly higher than other extracts. This can be due to the release of phenolic compounds from the cell matrix during the drying process or may occur during the processes used on the mushrooms that accelerate the release of bound phenolic compounds by the breakdown of cellular constituents. They also reported a positive correlation between antioxidant activity and the phenolic content by the method applied to the extracts.

### 4.3 pH and acidity

As expected, the results showed increased acidity and decreased pH in yogurts during the storage time. The possible reason could return to the type of prebiotic which in turn stimulate the metabolic activity of probiotics and production of organic acids during storage (Milani & Koocheki, 2011). In addition, production of lactic acid by starter culture of yogurt (*Streptococcus thermophilus* and *Lactobacillus bulgaricus*) should not be ignored during fermentation (Sekhavatizade, Karami, Savand, & Sadeghi, 2015). It is believed that supportive effects of β‐glucan of *Auricularia auricular* on the growth of starter culture and probiotics cause production of lactic and propionic acids which is consistent with the results of present study and Vasiljevic et al. (2007). In another study, the reason for increased acidity was reported as Bifidobacterium utilizes the complex polysaccharides and produces various organic acids (Van der Meulen, Avonts, & De Vuyst, 2004).

### 4.4 Syneresis

In this study, the amount of syneresis increased during storage. In fact, the amount of syneresis affecte by the type of prebiotic and the storage time. The possible reason for the increased syneresis could be related to protease enzymes present in AA. These enzymes coagulate the casein of milk and increase the amount of released serum. These findings support Raofi Asl Soofiani (2018) research results which reported the presence of proteases in the *Auricularia auricular* mushroom for higher syneresis. These compounds curdle or break the structure of casein of milk. The similar results were reported by Vasiljevic et al. (2007). Based on their findings, the addition of β‐glucan extracted from oat and barley to yogurts caused higher syneresis compared to inulin containing and control group yogurts. They reported weakness of the gel structure in the presence of polysaccharides as a cause of higher syneresis. Another study reported formation of 2 layer as a result of thermodynamic incompatibility between milk proteins and added polysaccharides for increased syneresis (Tolstoguzov, 2003). Another reason for syneresis could be the activity of lactic acid bacteria which reach the pH of milk casein to its isoelectric point and cause syneresis of yogurts (Vital et al., 2015).

### 4.5 Sensory evaluation

The results of present study showed that probiotic yogurt containing AAE had lower acceptance compared to control and probiotic yogurts. Furthermore, concentration‐dependent effect on sensory evaluation was observed as the higher concentration of AAE the lower overall acceptance. The possible reason for lower acceptance could be the bitter taste caused by extensive proteolysis effect of proteases present in AA. These enzymes lead to excessive proteolysis of proteins and finally production of short‐chain peptides which cause bitter taste in yogurts. On the other hand, higher syneresis also affects negatively the appearance of yogurt as panelists scored lower AAE containing yogurts compared to control group. The concentration‐dependent effect was also reported by Fan et al. (2007). Based on their results, the use of flour containing AA up to 9% concentration did not affect adversely the sensory properties. But, the higher concentration (12%) caused the lowest score for sensory properties (aroma, texture, taste, and mouth feel). Thus, upon development of any food product with AAE, the concentration of AAE should be optimized so as to achieve both the higher health benefits and sensory properties.

## 5 CONCLUSION

In the present study, two concentrations of *Auricularia auricula* extract (0.05% and 0.1%) were used and functional and sensorial properties of yogurt were investigated during 28 days of storage at 4^O^C. The survival of *L. acidophilus La‐5* and *B. bifidum Bb‐12* in the presence of 0.05% and 0.1% AAE in synbiotic yogurt has been improved about 0.35 and 0.43 log CFU/g and 0.58 and 0.51 log CFU/g, respectively. Furthermore, the antioxidant activity and total phenolic content have been increased to 115.30 mg BHT eq./kg and 1,057.6 mg Gallic acid/kg after 28 days of storage, respectively. Physiochemical properties of produced yogurts were not altered significantly (*p* < .05). However, significant difference was observed in terms of sensorial parameters (*p* > .05). Moreover, concentration‐dependent effect of AAE on sensory properties was also observed as the increased concentration of AAE the decreased score of sensory parameters. Thus, based on results 0.05% AAE is recommended for its good prebiotic activity and better sensory properties.

## CONFLICT OF INTEREST

The authors declare that there are no conflicts of interest.

## ETHICAL STATEMENT

This study does not involve any human or animal testing.
